# Purification and Characterization of His-Tagged Recombinant *Bacteroides fragilis* Toxin-2 Variants In Vitro and In Vivo

**DOI:** 10.3390/toxins18040189

**Published:** 2026-04-16

**Authors:** Woo-Seung Kim, Soohyun Lee, Ki-Ju Kwon, So-Min Kim, Ki-Jong Rhee

**Affiliations:** 1 Department of Biomedical Laboratory Science, College of Software Digital Healthcare Convergence, Yonsei University at MIRAE Campus, Wonju 26493, Republic of Korea; redberry1245@yonsei.ac.kr (W.-S.K.); dontgijup@yonsei.ac.kr (K.-J.K.); soming@yonsei.ac.kr (S.-M.K.); 2 Molecular Phytobacteriology Laboratory, Infectious Disease Research Center, Korea Research Institute of Bioscience and Biotechnology (KRIBB), Daejeon 34141, Republic of Korea; ysh76@kribb.re.kr

**Keywords:** enterotoxigenic *Bacteroides fragilis*, *B. fragilis* toxin, BFT receptor

## Abstract

*Bacteroides fragilis* is a major commensal bacterium of the human colon. However, enterotoxigenic *B. fragilis* (ETBF) secretes *B. fragilis* toxin (BFT), a zinc-dependent metalloprotease that cleaves E-cadherin and promotes chronic inflammation and colorectal tumorigenesis. Despite extensive research, the cellular receptor for BFT remains unidentified. In this study, we developed His-tagged recombinant BFT variants including both catalytically active and inactive forms to facilitate biochemical and functional analyses. Functional assays confirmed that the active variant retained proteolytic activity and induced characteristic cellular responses, while the inactive variant served as an effective negative control. These results establish a robust experimental platform for BFT receptor identification and mechanistic studies of BFT-host interactions. The active and inactive BFT variants provide essential molecular tools for investigating ETBF pathogenicity and developing therapeutic interventions.

## 1. Introduction

The human gastrointestinal tract harbors approximately 100 trillion microorganisms forming a complex ecosystem essential for nutrient metabolism, immune regulation, and pathogen defense [1,2]. Among these, the genus Bacteroides constitutes approximately 30% of the colonic microbiota, representing one of the most abundant and functionally significant bacterial groups [3,4]. *B. fragilis*, accounting for 1–2% of the intestinal microbiota, holds considerable biological importance due to its unique immunomodulatory properties and dual role as both beneficial commensal and opportunistic pathogen. The beneficial aspects of *B. fragilis* colonization are well documented. This bacterium produces polysaccharide A (PSA), a unique carbohydrate antigen presentable by MHC class II molecules. PSA presentation by dendritic cells induces differentiation of regulatory T cells (Tregs) producing anti-inflammatory cytokine IL-10, preventing experimental colitis and correcting systemic immune imbalances [5,6]. However, *B. fragilis* possesses a paradoxical dual nature. While most strains are non-pathogenic (nontoxigenic *B. fragilis*, NTBF), a subset secretes *B. fragilis* toxin (BFT) and is classified as enterotoxigenic *B. fragilis* (ETBF) [7]. Global ETBF prevalence varies considerably, with detection rates of 10–20% in the general population. Strikingly, ETBF prevalence is substantially elevated in inflammatory bowel disease (IBD) patients, with rates of 38–50% compared to 10–20% in healthy controls [8].

BFT is a 20 kDa zinc-dependent metalloprotease exerting pleiotropic effects on intestinal epithelial cells [9]. BFT belongs to the metzincin superfamily, characterized by a conserved zinc-binding motif (HEXXHXXGXXH) coordinating a catalytic zinc ion essential for proteolytic activity [10]. The bft gene encodes a 44 kDa immature precursor (pro-BFT) that is proteolytically processed by fragipain to generate the 20 kDa mature, catalytically active BFT. BFT is not retained within the periplasmic space but is secreted as an extracellular exotoxin. It has been reported that BFT is secreted via outer membrane vesicles (OMVs), 20–200 nm spherical bilayered structures protecting BFT from degradation and enabling long-distance delivery [11]. The well-known biological activity of primary BFT is a cleavage of E-cadherin, a 120 kDa transmembrane glycoprotein serving as the structural and functional cornerstone of adherens junctions [12,13,14]. BFT specifically cleaves extracellular domain of E-cadherin, disrupting the adhesive interface [12]. A critical consequence is liberation of β-catenin from junctional complexes. Under homeostatic conditions, cytoplasmic β-catenin is targeted for degradation by the destruction complex, maintaining low nuclear β-catenin levels. However, BFT-induced E-cadherin cleavage releases junctional β-catenin into the cytoplasm, overwhelming the destruction complex. Stabilized β-catenin translocates to the nucleus, binding TCF/LEF transcription factors and activating oncogenes including c-Myc and cyclin D1.

Beyond E-cadherin cleavage, BFT activates multiple signaling cascades. The NF-κB pathway is rapidly activated, inducing transcription of pro-inflammatory cytokines, most notably IL-8 (human) and CXCL1/KC (mouse) [15,16]. These chemokines induce massive neutrophil infiltration into the intestinal mucosa [17]. The STAT3 pathway is also activated, playing a critical role in oncogenic effects. BFT induces STAT3 phosphorylation at tyrosine 705, promoting expression of genes involved in cell survival, proliferation, angiogenesis, and immune evasion. Recent studies revealed that BFT induces a specific Th17 immune response [18]. BFT-stimulated cells secrete IL-6, IL-23, and IL-1β, driving naïve CD4+ T cell differentiation into Th17 cells [19]. The sustained Th17 response creates chronic inflammatory conditions strongly implicated in both IBD and CRC pathogenesis.

The sole genetic determinant distinguishing ETBF from NTBF is the presence or absence of the *B. fragilis* pathogenicity island (BfPAI) [20]. The BfPAI contains not only the *bft* gene but also encodes metalloprotease II (*mpII*). The BfPAI exhibits characteristic features of mobile genetic elements, with 12-base pair direct repeats at both ends and consistent insertion at the same genomic location in all ETBF strains examined. Critically, the BfPAI resides within a larger conjugative transposon designated CTn86, providing compelling evidence that ETBF strains acquired the pathogenicity island through horizontal gene transfer [21]. Whole-genome sequencing revealed that ETBF strains do not form a monophyletic clade, consistent with multiple independent BfPAI acquisition events. Three *bft* gene variants have been identified, *bft-1*, *bft-2*, and *bft-3* [21]. Despite sharing approximately 80–85% nucleotide sequence identity, the variants exhibit distinct biological properties. BFT-2 is the most potent variant, inducing more rapid and severe E-cadherin cleavage and inflammatory responses [22]. In germ-free mouse models, BFT-2-producing ETBF strains induce more severe colitis with greater epithelial damage and higher pro-inflammatory cytokine levels. Epidemiologically, *bft-1* is most prevalent globally (60–70% of isolates), *bft-2* represents 20–30%, while *bft-3* exhibits geographical restriction, being found predominantly in Southeast Asia [23].

To facilitate mechanistic studies, His-tagged recombinant BFT-2 variants, including catalytically active and inactive forms, provide a powerful platform for investigating toxin–host interactions. These variants enable the identification of host cell receptors through affinity-based approaches and allow discrimination between proteolysis-dependent and -independent cellular responses.

BFT receptor (BFTR) identification is essential for understanding ETBF pathogenic mechanisms and developing targeted therapeutics [24]. However, BFTR identification has proven extraordinarily challenging, primarily due to difficulties in obtaining purified, active BFT [19]. Previous studies predominantly used conditioned medium from ETBF cultures containing secreted BFT along with numerous other bacterial products and residual medium components. This approach suffered from critical limitations. First, the masking effect, brain-heart infusion broth supplemented with hemin and vitamin K (BHIS) contains complex protein mixtures that physically obstruct BFT-cell interactions [25]. Second, BFT quantification was impossible without specific antibodies. Third, biochemical studies were severely constrained, as receptor identification strategies such as pull-down assays, co-immunoprecipitation, and surface plasmon resonance require purified proteins [26]. To overcome these limitations, the present study aimed to develop *B. fragilis* strains producing His-tagged recombinant BFT (hBFT). The His-tag exhibits high-affinity binding to immobilized nickel or cobalt ions, enabling one-step purification via immobilized metal affinity chromatography (IMAC). IMAC typically achieves > 90% purity in a single step with 50–80% yields, dramatically outperforming conventional approaches [27,28,29,30]. His-tagged BFT provides multiple advantages, such as high purity eliminating masking effects, direct detection and quantification using commercial anti-His antibodies, utility in pull-down assays and co-immunoprecipitation, application in biophysical techniques for measuring binding kinetics, facilitation of structural studies, and development of neutralizing antibodies. The first objective was to develop *B. fragilis* strains (His-rNTBF and His-rETBF) producing His-tagged BFT and obtain purified hBFT with preserved biological activity.

This study represents a significant methodological advancement in ETBF research through the development of His-tagged BFT constructs. Both active His-tagged BFT-2 (from His-rETBF) and inactive His-tagged BFT-2 (from His-rNTBF) were successfully generated in *B. fragilis*, establishing a rigorous experimental system capable of clearly distinguishing BFT-specific effects from non-specific background effects [17]. This dual-construct approach addresses a critical gap in previous ETBF studies, where the absence of appropriate negative controls often confounded interpretation of experimental results. The purified hBFT provides essential molecular tools for diverse downstream applications including receptor identification studies, BFT functional characterization, neutralizing antibody development, structural biology investigations, and high-throughput screening of BFT inhibitors. These developments lay important groundwork for future BFTR identification efforts by narrowing the candidate pool and refining experimental strategies.

## 2. Results

### 2.1. Strategic Cloning Design and PCR Amplification

The development of His-tagged BFT constructs required strategic placement of a 6× His tag at the C-terminus of the mature BFT domain (amino acids 220–405) to preserve protein functionality while facilitating purification through immobilized metal affinity chromatography. The 1218 bp *bft-2* gene encodes a precursor protein with a critical fragipain cleavage site between Arg219 and Ala220 that demarcates the transition from the N-terminal secretion signal to the mature C-terminal toxin domain.

A two-step PCR strategy was employed to generate the required molecular constructs. pFD340-*bft-2* (10,742 bp) served as template, containing the complete *bft-2* sequence within a suitable expression vector backbone. The first-round PCR incorporated both a *Sma*I restriction site for directional cloning and a 6× His tag while maintaining the integrity of the BFT-2 coding sequence. Specifically designed primers introduced a *Sma*I restriction site at the 5′ terminus and incorporated a 6× His sequence followed by an appropriate stop codon at the 3′ terminus. This approach generated a 177 bp amplification product, with agarose gel electrophoresis confirming the correct size and purity.

The second-round PCR represented a critical refinement step to optimize the molecular construct for efficient directional cloning. This additional amplification introduced complementary restriction sites that enabled precise, directional insertion into the target vector while maintaining the previously incorporated His-tag sequence. Addition of *Bam*HI restriction sites alongside the existing *Sma*I sites provided optimal cloning flexibility and ensured correct insert orientation during ligation. The 177 bp product from the first-round PCR served as template DNA, with primers that embedded both *Sma*I and *Bam*HI restriction sites while preserving the internal His-tag sequence and maintaining proper reading frame alignment. This strategy yielded a 232 bp product representing the complete insert DNA with optimized restriction sites, with size verification through agarose gel electrophoresis demonstrating the appropriate molecular weight increase consistent with the addition of restriction site sequences.

### 2.2. Restriction Enzyme Digestion and Ligation Optimization

The restriction enzyme digestion strategy employed double digestion with *Sma*I and *Bam*HI enzymes to generate compatible cohesive ends that enabled directional, high-efficiency ligation while preventing vector self-ligation or incorrect insert orientation. Simultaneous digestion with both enzymes created precisely defined sticky ends that facilitated efficient ligation of the 232 bp insert into the linearized pFD340-bft-2 vector backbone.

Optimization of digestion reaction timing emerged as a critical parameter for maximizing ligation efficiency. Direct comparison between 2 h and 24 h digestion protocols, maintaining identical enzyme concentrations and reaction conditions, demonstrated that 24 h incubation achieved significantly greater digestion efficiency compared to the 2 h protocol, with enhanced completeness of restriction enzyme cleavage and improved substrate quality. Based on these optimization results, the 24 h digested samples were selected for subsequent ligation reactions.

T4 DNA ligase-mediated ligation assembled recombinant constructs by combining the double-digested vector DNA with the processed insert DNA in appropriate molar ratios to favor insert incorporation over vector self-ligation. Systematic evaluation of different ligation conditions, varying parameters such as insert-to-vector molar ratios and DNA concentrations, revealed that optimized conditions produced enhanced efficiency in construct assembly compared to alternative conditions. This optimization identified the ideal ligation parameters for generating high-quality pFD340-*bft-2*(His_6_) constructs. The construction process for pFD340-*bft-2*(E349A-His_6_) to generate I-hBFT followed identical procedures except for the utilization of pFD340-*bft-2*(E349A) as template DNA.

### 2.3. Transformation and Conjugation-Mediated Transfer

Initial transformation into *E. coli* TOP10 competent cells served as the primary step for construct validation and long-term storage of the recombinant plasmids. Heat shock transformation efficiently introduced the ligated constructs into competent cells, enabling initial propagation and validation. The transformation results demonstrated uptake and maintenance of the recombinant constructs in *E. coli* TOP10 cells, with recovered transformants carrying the correctly assembled His-tagged BFT constructs. Direct transformation attempts of *B. fragilis* NCTC9343 via heat shock and electroporation methods consistently failed across various experimental conditions tested. The conjugation-mediated transfer strategy represented a critical alternative approach for introducing His-pFD340 constructs into *B. fragilis*, based on its proven effectiveness for transferring plasmid DNA between bacterial species that are recalcitrant to direct transformation.

Conjugation between *E. coli* S17-1 λ pir donor strains carrying His-pFD340 construct and *B. fragilis* NCTC9343 recipient cells overcame the transformation barriers that prevented DNA uptake via direct methods. The methodology involved transformation of His-pFD340 plasmids into *E. coli* S17-1 λ pir donor strains, followed by mixing with wild-type *B. fragilis* recipient cells to facilitate optimal cell-to-cell contact conditions. Molecular confirmation of conjugation required rigorous analysis to verify plasmid transfer and eliminate false-positive results. Plasmid mini-prep analysis on isolated colonies provided molecular evidence of conjugation by demonstrating the presence of intact His-pFD340 plasmids in recipient *B. fragilis* cells. Analysis of six single colonies that emerged on selective medium, followed by plasmid DNA extraction and agarose gel electrophoretic analysis, confirmed plasmid size and integrity. Side-by-side electrophoretic comparison showed identical migration patterns for His-pFD340 plasmids isolated from both bacterial species, confirming structural conservation during the conjugation process.

### 2.4. Sequence Verification and Structural Analysis

Complete sequence verification of the engineered His-pFD340 constructs confirmed the precise molecular modifications introduced during the cloning process. Comprehensive sequencing of both pFD340-*bft-2*(His_6_) and pFD340-*bft-2*(E349A-His_6_) constructs ensured complete coverage of the modified regions. The sequencing results confirmed that both His-pFD340 constructs contained the 6× His codon correctly inserted upstream of the stop codon, while the pFD340-*bft-2*(E349A-His_6_) construct demonstrated the precise A-to-C nucleotide mutation at position 1070, resulting in the desired glutamic acid to alanine amino acid substitution.

Three-dimensional structural models of hBFT-2 variants were generated using AlphaFold3 based on complete sequences obtained from sequencing analysis. Structural visualization revealed distinct conformational features between wild-type and mutant variants, particularly highlighting the structural implications of the E349A substitution within the catalytic zinc-binding motif. The HEXXHXXXXXH zinc-coordinating sequence and its interaction with the metal cofactor were analyzed, revealing the structural basis for modulation of metalloprotease activity in the engineered BFT-2 constructs.

### 2.5. Protein Purification and Optimization

**Figure 1:**
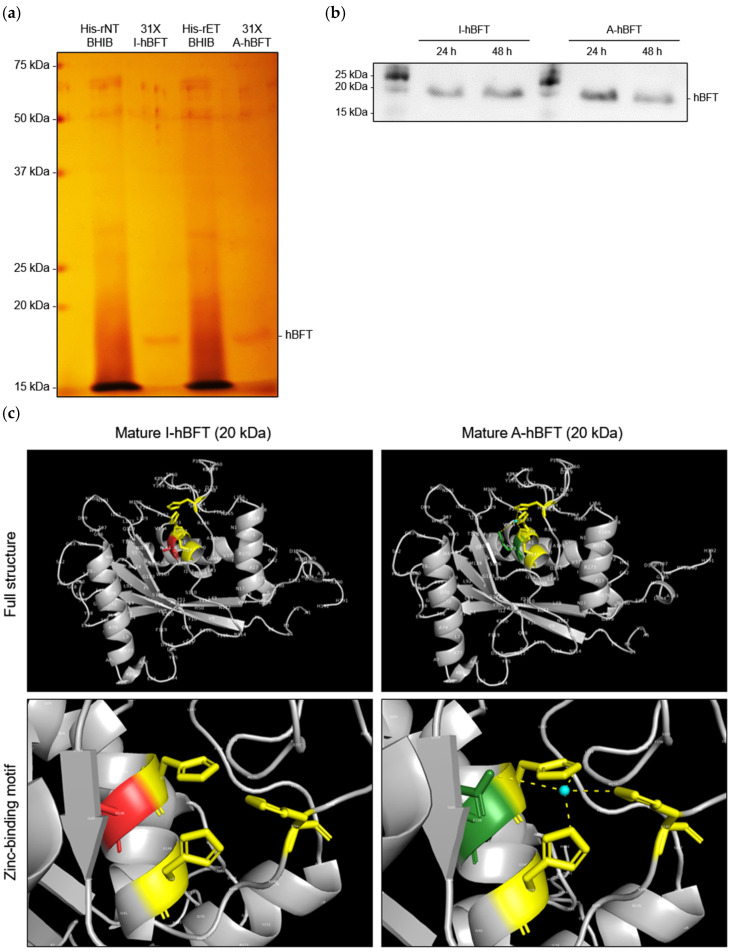
Purification and structural characterization of His-tagged BFT variants: (**a**) Silver-stained SDS-PAGE analysis of IMAC purification. Lane 1: His-rNTBF culture supernatant. Lane 2: Purified and concentrated I-hBFT. Lane 3: His-rETBF culture supernatant. Lane 4: Purified and concentrated A-hBFT. Purified hBFT samples show enrichment to near homogeneity with predominant bands at ~19 kDa. (**b**) Western blot analysis of culture duration effects on His-tagged BFT expression at 24 h and 48 h for I-hBFT (lanes 1–2) and A-hBFT (lanes 3–4). (**c**) AlphaFold3-predicted structures of mature I-hBFT and A-hBFT. The E349A mutation in I-hBFT disrupts the zinc-binding motif, highlighting structural basis for catalytic inactivation. Mutated alanine residue (A349) shown in red; original glutamic acid residue (E349) shown in green.

Validation of protein purification efficiency established reliable methods for obtaining pure His-tagged BFT suitable for functional assays and biochemical characterization. The combination of IMAC purification, concentration through microcentrifugal filter units, and diafiltration for imidazole removal effectively isolated His-tagged BFT from complex bacterial culture supernatants, yielding pure protein preparations suitable for detailed biochemical and functional studies. Cultivation of His-rNTBF and His-rETBF strains in BHIB medium, followed by IMAC purification of culture supernatants using nickel-affinity chromatography, 31-fold concentration via microcentrifugal filter units, and diafiltration to remove imidazole, with final protein purity assessed through SDS-PAGE and sensitive silver staining detection revealed that BHIB culture supernatants from His-rNTBF and His-rETBF contained multiple protein bands as expected for complex bacterial secretions, while both inactive His-tagged BFT (I-hBFT) and active His-tagged BFT (A-hBFT) samples subjected to the complete IMAC purification, concentration, and diafiltration protocol displayed a predominant band at ~19 kDa, consistent with hBFT, accompanied by minor higher-molecular-weight bands (~50–70 kDa), indicating partial co-purification of additional proteins (Figure 1a).

Resolution of technical challenges associated with imidazole interference in protein analysis ensured optimal experimental conditions for future biochemical studies. The band smearing observed during SDS-PAGE analysis of His-tagged BFT in imidazole-containing elution buffer could be eliminated through alternative sample preparation methods that maintained protein integrity while improving electrophoretic resolution. Systematic comparisons of different sample preparation approaches, including standard IMAC elution in imidazole buffer, complete diafiltration for imidazole removal, and heat treatment at 70 °C for 5 min as an alternative method to reduce imidazole interference without requiring extensive buffer exchange procedures, confirmed that His-tagged BFT eluted in imidazole buffer consistently exhibited band smearing that compromised gel quality, while samples subjected to diafiltration for complete imidazole removal displayed sharp, well-defined bands without smearing artifacts. Additionally, heat treatment at 70 °C for 5 min provided an effective alternative approach for improving band resolution in imidazole-containing samples.

Investigation of culture duration effects on protein expression levels optimized bacterial cultivation conditions for consistent His-tagged BFT production. Examination of temporal expression patterns revealed that bacterial protein secretion levels can vary significantly with culture duration due to factors such as growth phase dynamics, nutrient depletion, protein degradation, and metabolic changes. Parallel cultivation of His-rNTBF and His-rETBF strains for either 24 or 48 h under standardized conditions, followed by His-tagged BFT purification and quantitative Western blot analysis to assess relative protein levels, revealed distinct temporal expression patterns for the two BFT variants, with inactive His-tagged BFT (I-hBFT) showing equivalent band intensities between 24 h and 48 h culture periods, indicating stable expression and accumulation over this timeframe, while active His-tagged BFT (A-hBFT) demonstrated significantly stronger band intensity at 24 h compared to 48 h, suggesting potential degradation or reduced stability of the active toxin variant during extended cultivation (Figure 1b).

### 2.6. Functional Characterization and Clone Selection

**Figure 2:**
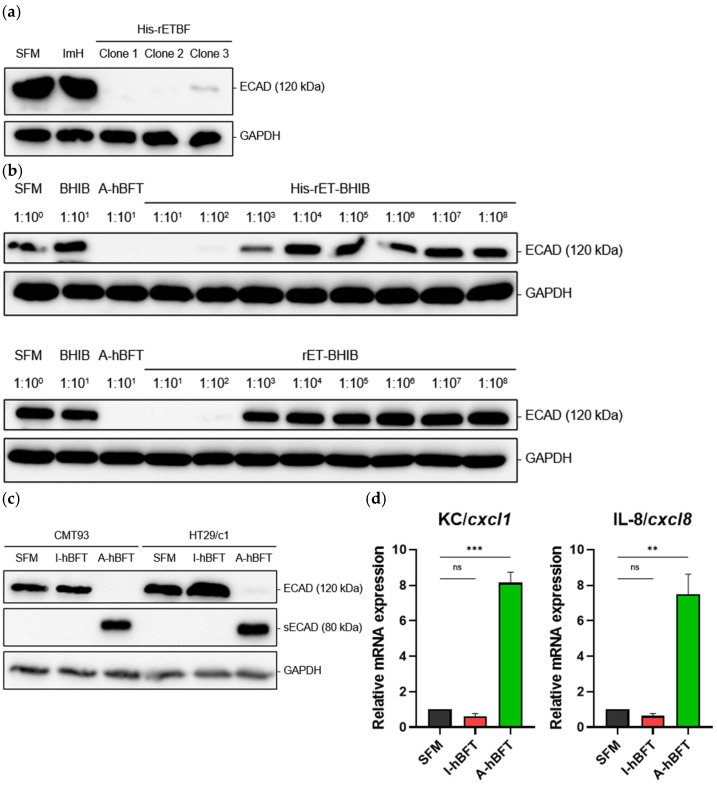
Functional characterization of His-tagged BFT variants: (**a**) E-cadherin cleavage activity of A-hBFT from three His-rETBF clones. A-hBFT cleaves full-length E-cadherin (120 kDa) while serum-free medium (SFM) and imidazole controls do not. Clone 1 was selected for subsequent experiments. (**b**) Serial dilution analysis (1:10^1^ to 1:10^8^) comparing biological activity of His-tagged and wild-type BFT culture supernatants. (**c**) Cross-species validation in mouse (CMT93) and human (HT29/c1) epithelial cells. A-hBFT cleaves E-cadherin in both cell types while I-hBFT serves as negative control. Soluble 80 kDa E-cadherin ectodomain (sECAD) appears in culture supernatants following A-hBFT treatment. (**d**) RT-qPCR analysis of inflammatory cytokine response. A-hBFT significantly upregulates *KC*/*cxcl1* (CMT93) and *IL-8*/*cxcl8* (HT29/c1) mRNA expression. Data represent mean ± SEM. ** *p* < 0.01, *** *p* < 0.001. ns = not significant.

Functional characterization of recombinant *B. fragilis* clones validated the biological activity of His-tagged BFT variants and assessed clone-to-clone variability in protein function. Evaluation of E-cadherin cleavage activity represented the gold standard for assessing BFT biological function, as E-cadherin cleavage is the primary mechanism through which BFT exerts its pathogenic effects on intestinal epithelial cells. Cultivation of three selected His-rETBF clones in BHIB medium, followed by purification of secreted His-tagged BFT from culture supernatants using immobilized metal affinity chromatography, protein quantification via BCA assay, and standardized dilution to equivalent concentrations before application to HT29/c1 epithelial cells for E-cadherin cleavage assessment revealed that clones 1 and 2 exhibited complete cleavage of full-length E-cadherin when applied to HT29/c1 cells, demonstrating robust biological activity of the His-tagged BFT variants, while clone 3 showed diminished E-cadherin cleavage activity despite treatment with equivalent BFT concentrations (Figure 2a).

Assessment of potential interference from purification reagents on biological activity ensured that experimental conditions accurately reflected protein-specific effects rather than artifacts from purification components. Treatment of HT29/c1 epithelial cells with various experimental conditions including imidazole alone, active His-tagged BFT alone, and combinations of imidazole with active His-tagged BFT, followed by assessment of E-cadherin cleavage through standard Western blot analysis demonstrated that HT29/c1 cells treated with imidazole alone exhibited E-cadherin band intensity identical to cells treated with serum-free medium, confirming that imidazole had no direct effects on E-cadherin levels or cellular integrity, while active His-tagged BFT completely cleaved E-cadherin regardless of imidazole presence.

Comparative assessment of biological activity between His-tagged and wild-type BFT validated that the introduced molecular modifications did not compromise protein function (Figure 2b). Cultivation of equal numbers of His-rETBF and rETBF bacterial cells, collection of BHIB culture supernatants, serial dilution of both supernatants at 1:10 ratios in serum-free DMEM medium, and application to HT29/c1 epithelial cells for one hour under standardized conditions, followed by assessment of E-cadherin cleavage through Western blot analysis revealed that at 1:10^3^ dilution, cells treated with His-rETBF BHIB supernatant exhibited weaker E-cadherin band intensity compared to cells treated with rETBF BHIB supernatant, indicating that the His-tagged variant actually cleaved E-cadherin more effectively than the wild-type protein.

### 2.7. Cross-Species Validation and Mechanistic Studies

Cross-species validation of His-tagged BFT biological activity established the universal applicability of the recombinant protein across different mammalian epithelial cell systems. Parallel treatment of HT29/c1 human epithelial cells and CMT93 mouse epithelial cells with serum-free DMEM as vehicle control, I-hBFT, or A-hBFT at standardized concentrations of 100 ng/mL for 3 h at 37 °C, followed by comprehensive assessment of cellular responses through Western blot analysis revealed that serum-free DMEM vehicle control treatments produced no significant E-cadherin cleavage in either cell line, confirming the specificity of observed effects to BFT treatment, while A-hBFT treatment resulted in complete cleavage of full-length E-cadherin in cell lysates from both CMT93 and HT29/c1 cells (Figure 2c).

Detection and characterization of soluble E-cadherin cleavage products provided mechanistic insights into BFT-mediated cellular effects and confirmed the proteolytic specificity of the recombinant protein. Collection of culture supernatants from HT29/c1 and CMT93 cells following treatment with vehicle control, I-hBFT, or A-hBFT under standardized conditions, followed by Western blot analysis specifically targeting the soluble E-cadherin fragments using appropriate antibodies confirmed the presence of soluble 80 kDa E-cadherin fragments in samples from both CMT93 and HT29/c1 cells treated with A-hBFT, demonstrating that the His-tagged BFT successfully induced proteolytic cleavage and release of E-cadherin fragments with the expected molecular characteristics.

Assessment of inflammatory cytokine responses evaluated the downstream cellular consequences of BFT treatment and validated the physiological relevance of His-tagged BFT-induced cellular responses (Figure 2d) [16]. Quantitative PCR analysis of inflammatory cytokine mRNA expression in HT29/c1 and CMT93 cells following treatment with vehicle control, I-hBFT, or A-hBFT under standardized conditions, with specific primer sets designed to detect human IL-8 and mouse KC (CXCL1) transcripts revealed that A-hBFT treatment induced a 7-fold increase in IL-8 mRNA levels in HT29/c1 cells, consistent with previous reports of BFT-induced inflammatory responses, while CMT93 cells exhibited an 8-fold upregulation of KC (CXCL1) mRNA compared to negative controls.

### 2.8. Receptor-Mediated BFT Binding and Cellular Trafficking

**Figure 3:**
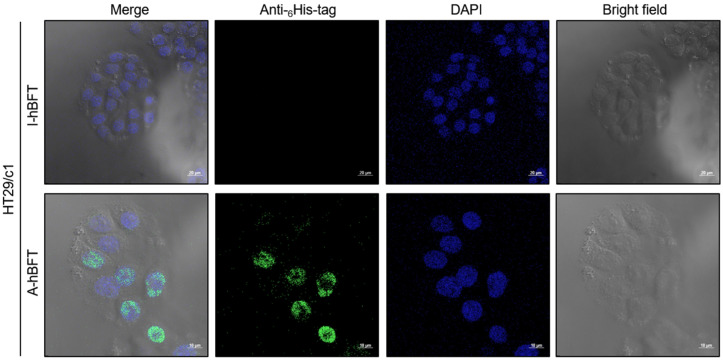
Cellular binding and trafficking of His-tagged BFT variants: Confocal microscopy of HT29/c1 cells treated with I-hBFT or A-hBFT for 30 min. Green fluorescence indicates anti-6× His tag detection; blue shows DAPI-stained nuclei. I-hBFT shows no detectable cellular binding, while A-hBFT demonstrates clear binding with apparent intracellular trafficking. The E349A mutation in I-hBFT abolishes cellular binding capacity. Scale bar = 20 μm.

Investigation of BFT cellular binding mechanisms revealed critical insights into the relationship between biological activity and receptor binding. The BFT derived from rNTBF harbors an E349A mutation that abolishes its biological activity, resulting in the absence of BFT-induced biological activities including E-cadherin cleavage, IL-8 secretion, and cell rounding in HT29/c1 cells. To elucidate whether the E349A mutation eliminates receptor binding capacity, HT29/c1 human intestinal epithelial cells were cultured and treated with either I-hBFT or A-hBFT for 30 min at 37 °C. Following treatment, cells were fixed and immunostained with anti-6× His tag primary antibody and Alexa Fluor 488-conjugated secondary antibody to detect the His-tagged BFT variants, with nuclei counterstained with DAPI. The results demonstrated that HT29/c1 cells treated with I-hBFT exhibited no detectable fluorescence signal, while HT29/c1 cells treated with A-hBFT displayed clear fluorescence signals, with the notable observation that fluorescence was localized within the nuclear region (Figure 3).

Investigation of BFT responsiveness across different mouse intestinal epithelial cell lines provided evidence for the requirement of a specific BFT receptor (BFTR). While mouse intestinal epithelial cell lines are generally known to be unresponsive to BFT, showing no characteristic biological responses such as E-cadherin cleavage, cytokine secretion, or cell rounding, the mouse colon epithelial cell line CMT93 represents a notable exception as the first and only BFT-responsive mouse cell line identified to date. Three mouse cell lines were cultured: the BFT-responsive CMT93 and the BFT non-responsive MSIE and YAMC cell lines. Cells were treated with serum-free medium (SFM), I-hBFT, or A-hBFT at 37 °C for 5 min, followed by immunofluorescence staining using anti-6× His tag as the primary antibody.

**Figure 4:**
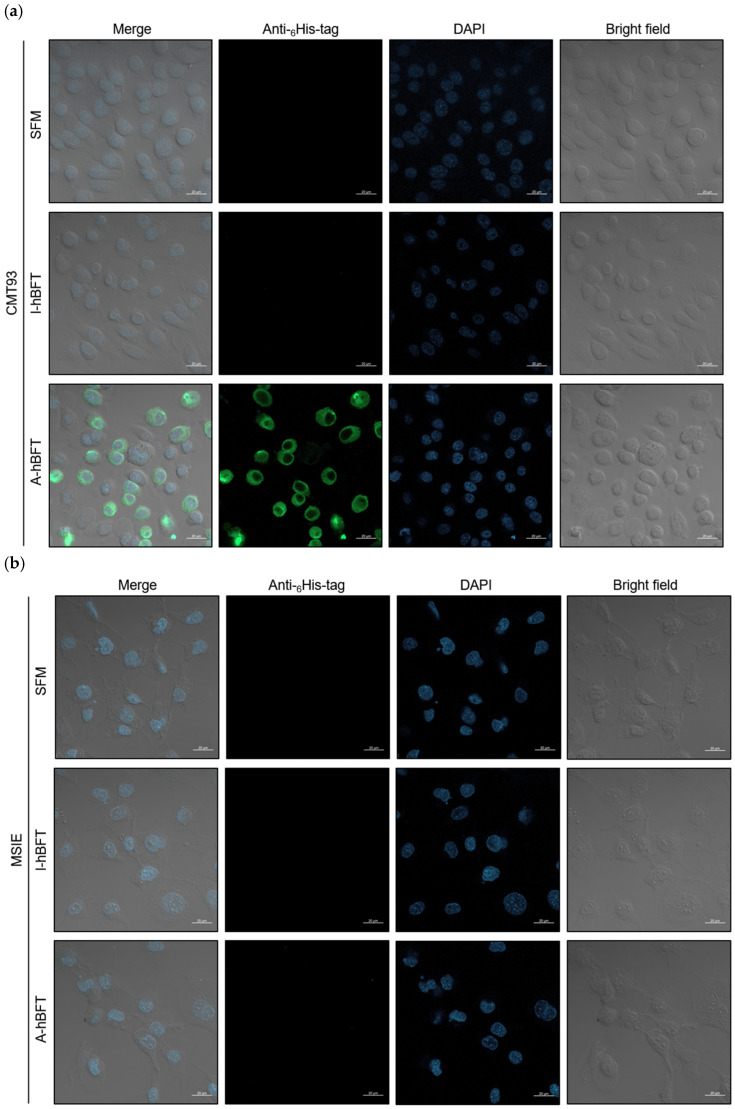

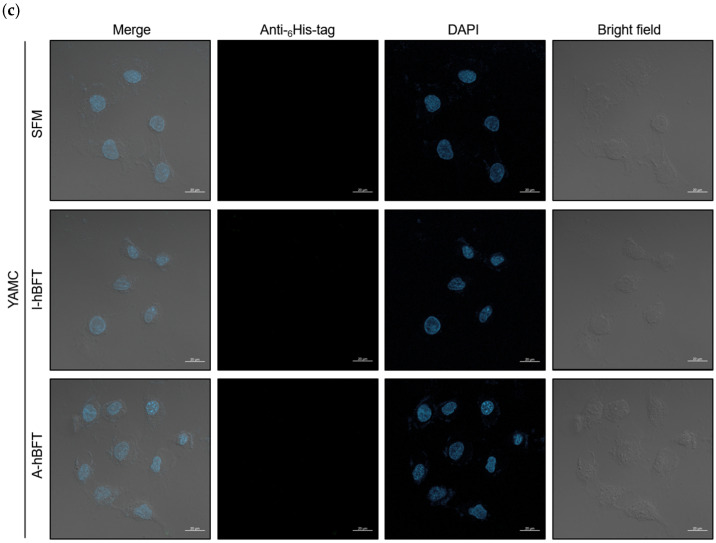
BFT receptor-dependent cellular binding patterns across mouse intestinal epithelial cell lines: Confocal microscopy of mouse intestinal epithelial cells treated with SFM, I-hBFT, or A-hBFT. Green fluorescence indicates hBFT detection via anti-6× His tag. (**a**) CMT93 cells (BFT-responsive) show binding only with A-hBFT treatment; (**b**) MSIE cells (BFT-non-responsive) show no binding under any condition; (**c**) YAMC cells (BFT-non-responsive) similarly show no binding signals. Differential binding patterns suggest BFT responsiveness depends on specific receptor presence rather than direct E-cadherin interaction. Scale bar = 20 μm.

The results supported the hypothesis that BFT non-responsive cells lack BFTR and therefore cannot bind hBFT (Figure 4). CMT93 cells treated with SFM or I-hBFT exhibited no fluorescence, while those treated with A-hBFT showed clear fluorescence signals, confirming specific binding of the active toxin. In contrast, both MSIE and YAMC cells showed no fluorescence regardless of treatment condition (SFM, I-hBFT, or A-hBFT), indicating the absence of hBFT binding. Extended incubation for 30 min confirmed these findings, with CMT93 cells treated with A-hBFT resulting in clear fluorescence signals, while both SFM control and I-hBFT treatment showed no fluorescence. MSIE cells exhibited no fluorescence under any treatment conditions, including A-hBFT (Figure 4b). Similarly, YAMC cells showed no fluorescence signals regardless of treatment with SFM, I-hBFT, or A-hBFT (Figure 4c).

### 2.9. In Vivo Characterization

In vivo validation of His-recombinant *B. fragilis* was conducted using a well-established mouse infection model. Female C57BL/6 mice (10 weeks old) were randomly assigned to four groups: Mock control (antibiotic treatment without bacterial challenge), His-rNTBF infection (non-toxigenic control), His-rETBF infection (His-tagged toxigenic strain) and rETBF infection (conventional toxigenic strain). Following antibiotic pretreatment to reduce indigenous gut microbiota, mice received oral inoculation with the respective bacterial strains or sterile medium for 3 consecutive days.

**Figure 5:**
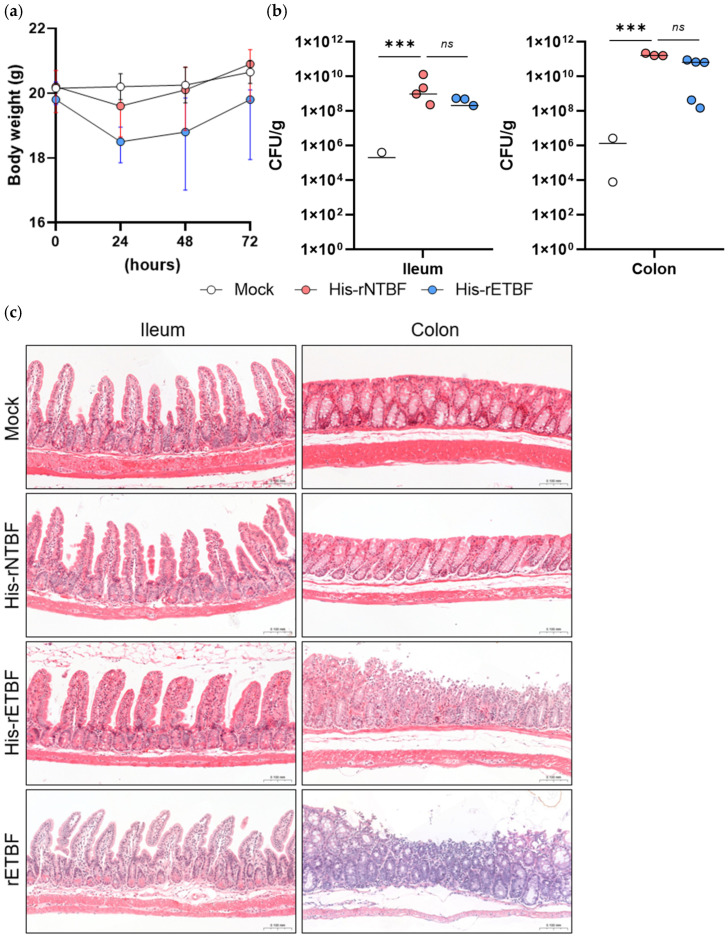
In vivo validation of His-rETBF pathogenicity in a mouse infection model: Female C57BL/6 mice were infected with Mock control (*n* = 2), His-rNTBF (*n* = 7), His-rETBF (*n* = 7), or rETBF (*n* = 5) for 3 days. (**a**) Body weight changes during infection. His-rETBF caused immediate weight loss while Mock and His-rNTBF groups maintained steady weight gain. Data represent median ± 95% CI. (**b**) Bacterial colonization (CFU/g) in small intestine and colon. All *B. fragilis* strains achieved robust colonization data represent median ± SEM; *** *p* < 0.001; ns, non-significant (**c**) Histopathological analysis (H&E staining) of ileum and colon sections. His-rETBF and rETBF induced characteristic colitis with epithelial shedding, crypt hyperplasia, and inflammatory infiltration, while Mock and His-rNTBF groups showed normal histology. Scale bar = 100 μm.

Validation of negative control conditions established baseline inflammatory status and confirmed the specificity of pathogenic effects observed in infected groups. Systematic histopathological examination of intestinal tissue sections from both ileum and colon of mice in the Mock control group (treated with antibiotics alone) and the His-rNTBF-infected group (infected with non-toxigenic recombinant *B. fragilis*), with tissue processing, sectioning, and staining performed according to standardized protocols, confirmed that both the Mock control group and His-rNTBF-infected group exhibited completely normal intestinal histology with no detectable signs of inflammation in either the ileum or colon, demonstrating preserved tissue architecture, intact epithelial barriers, and absence of inflammatory cell infiltration (Figure 5).

Monitoring of physiological parameters revealed distinct responses between toxigenic and non-toxigenic bacterial infections. Body weight measurements throughout the infection period demonstrated that His-rETBF-infected groups showed immediate weight loss following oral inoculation, with weight reduction becoming apparent within 24 h of the first bacterial challenge and persisting throughout the 3-day infection period (Figure 5a). In contrast, both Mock control and His-rNTBF groups maintained steady weight gain consistent with normal growth patterns in healthy mice, confirming that weight loss was specifically associated with toxigenic ETBF infection rather than bacterial colonization per se or experimental manipulation.

Bacterial colonization assessment confirmed successful establishment of *B. fragilis* strains in the gastrointestinal tract. Quantitative culture analysis of intestinal contents revealed robust bacterial colonization in both small intestine and colon for all *B. fragilis*-infected groups (Figure 5b). Colony-forming unit (CFU) counts demonstrated approximately 1 × 10^9^ CFU/g in small intestine samples and 1 × 10^11^ CFU/g in colon samples for His-rNTBF and His-rETBF groups, indicating that all recombinant strains maintained equivalent colonization capacity and that His-tagging did not affect bacterial fitness or intestinal colonization efficiency.

Comparative assessment of intestinal pathology between conventional His-rETBF and rETBF infection validated that the His-tagged variant retained equivalent pathogenic potential in vivo. Systematic infection studies with both His-rETBF and rETBF strains under identical experimental conditions, followed by comprehensive histopathological examination of both ileal and colonic tissue sections, revealed that both His-rETBF-infected and rETBF-infected groups exhibited identical patterns of intestinal pathology, with complete absence of inflammatory changes in the ileum but prominent colonic inflammation in both groups, perfectly recapitulating the established pattern of ETBF-induced colitis described in previous reports (Figure 5c).

Detailed characterization of specific histopathological features confirmed that His-rETBF induced the complete spectrum of pathological changes characteristic of ETBF-mediated colitis. The detailed histopathological examination confirmed the presence of all characteristic features of ETBF-induced colitis in His-rETBF-infected mice, including prominent epithelial cell layer shedding indicating direct BFT-mediated cytotoxic damage, significant crypt hyperplasia with markedly increased crypt thickness reflecting compensatory epithelial regeneration, and extensive neutrophil infiltration throughout the colonic mucosa demonstrating robust acute inflammatory responses. These findings provided comprehensive cellular and tissue-level validation that His-rETBF retained the complete pathogenic repertoire of conventional ETBF, inducing not only macroscopic inflammatory changes but also the full spectrum of microscopic pathological features that characterize BFT-mediated intestinal disease.

The anatomical specificity of inflammatory changes further confirmed the preserved tissue tropism of His-rETBF. Both His-rETBF and rETBF infections demonstrated anatomically restricted inflammatory changes affecting the colon while leaving the ileum completely unaffected, with comparable severity and distribution of pathological features between the two infection groups. This finding demonstrated that His-rETBF retained full colitogenic potential equivalent to conventional rETBF and confirmed that C-terminal His-tagging did not compromise any aspect of ETBF pathogenicity, including bacterial colonization efficiency, tissue tropism, or inflammatory response induction, thereby validating the His-tagged strain as a functionally equivalent substitute for conventional rETBF in experimental colitis models.

## 3. Discussion

This study addressed critical knowledge gaps in ETBF pathogenesis through systematic investigation. Both active and inactive forms of His-tagged BFT-2 were successfully developed, enabling high-purity hBFT purification while preserving biological activity. The development of both His-rETBF and His-rNTBF represents a technical advancement compared to previous studies that produced only active forms. The inactive BFT-2 serves as an ideal negative control for distinguishing BFT-specific effects from non-specific binding or experimental artifacts. The purified hBFT provides essential molecular tools for diverse downstream applications including BFT-specific antibody development, receptor identification studies, BFT functional characterization, neutralizing antibody development, structural biology investigations, and high-throughput screening of BFT inhibitors.

Cross-species validation demonstrated that A-hBFT retained full biological activity in both human (HT29/c1) and mouse (CMT93) epithelial cells, inducing complete E-cadherin cleavage and robust inflammatory cytokine responses. Specifically, A-hBFT treatment resulted in 7-fold and 8-fold upregulation of IL-8 and KC (CXCL1) mRNA levels in human and mouse cells, respectively, confirming that His-tagging does not compromise the toxin’s pro-inflammatory properties. This cross-species functionality validates hBFT as a universal research tool for both human disease modeling and animal studies.

After developing A-hBFT, the primary attempt was to identify the still-uncharacterized BFTR. Before attempting traditional co-immunoprecipitation (co-IP) methods, three experimental groups were prepared using untreated HT29/c1 cell lysates. The first group involved separating cell lysate by SDS-PAGE followed by transfer. The second group mixed cell lysate with hBFT before SDS-PAGE and transfer. The third group separated lysate by SDS-PAGE, transferred to nitrocellulose membrane, then treated the membrane with hBFT. All three groups were treated with primary antibody (mouse anti-His tag) and detection antibody (goat anti-mouse Ig) to observe whether BFTR bands could be detected. Multiple non-specific bands were observed without any meaningful bands presumed to be BFTR. This could suggest that BFTR does not bind BFT when denatured or isolated from cells, although numerous reasons could explain the failure to detect BFTR bands. This was concluded as an exploratory experiment.

In subsequent studies, methods of tagging BFT-2 with GFP, TurboID, or miniTurbo instead of His-tagging are being considered [31,32]. However, given that deletion of 8 amino acids from the C-terminal end of BFT results in loss of biological activity, attaching long tags is realistically difficult and requires methodological improvements [10,33,34]. 6× His tagging was selected because it represents the shortest length while efficiently enabling BFT separation. Proximity ligation assay (PLA) methods are also considered potentially useful for BFTR identification [35]. Using different primary antibodies for BFT and putative receptor proteins, when the two antibodies are within 30–40 nm proximity, DNA oligonucleotides linked to secondary antibodies become ligated. This ligated DNA is amplified by rolling circle amplification and becomes visible as fluorescent signal spots. Observing this amplified fluorescence by confocal microscopy to confirm co-localization on the cell surface would represent an excellent method for BFTR identification.

Regarding the apparent heterogeneity of BFT nuclear translocation in confocal images, careful examination of z-stack images revealed that all cells exhibited nuclear BFT accumulation. Time-course analysis revealed progressive A-hBFT internalization from initial membrane binding (1 min) to substantial nuclear accumulation (20–30 min), suggesting an active cellular uptake mechanism rather than passive diffusion. Single z-plane images appeared heterogeneous due to varying cell heights and nuclear positions in HT29/c1 cultures. Whether BFT functions as a transcription factor in the nucleus remains an intriguing question requiring nuclear fractionation studies and chromatin immunoprecipitation experiments. While β-catenin is known to activate inflammatory cytokine genes, direct transcriptional roles for BFT, if any, remain uncharacterized [36,37].

Although not common, toxins translocating to the nucleus and functioning directly have been reported. In the Shiga toxin family, some Stx2 variants have been reported to undergo retrograde transport through Golgi, endoplasmic reticulum, cytosol, and nucleus pathways, with nuclear entry aimed at host DNA damage and cell cycle inhibition [38]. Although BFT shows different biological activity from Shiga toxin by increasing cell proliferation in HT29/c1, the nuclear translocation pathway provides valuable reference information. Additionally, nucleolar localization of *Clostridioides difficile* transferase (CDT) and some fragments of pertussis toxin have been reported [39,40,41,42,43]. Direct evidence that BFT translocates to the nucleus requires observation by Western blot after nucleus fractionation, though current experimental evidence consists only of colocalization spots visible by confocal microscopy. If nuclear localization is verified in future studies, transcriptional profiling and nuclear localization signal (NLS) mutant experiments would be needed to elucidate nuclear functions of BFT.

The differential responsiveness observed among mouse intestinal epithelial cell lines provides compelling evidence for BFTR specificity. While CMT93 cells (the only known BFT-responsive mouse cell line) showed clear A-hBFT binding, the BFT-non-responsive MSIE and YAMC cell lines exhibited no hBFT binding regardless of treatment duration or toxin activity status. This pattern strongly supports the hypothesis that BFT responsiveness is determined by the presence or absence of specific cellular receptors rather than universal E-cadherin availability, reinforcing the concept that BFTR identification is crucial for understanding ETBF pathogenesis mechanisms.

His-rNTBF secreting I-hBFT was initially developed with the hypothesis that I-hBFT might bind BFTR without inducing cleavage, thus serving as a competitive inhibitor for therapeutic applications [44,45]. However, confocal microscopy revealed that I-hBFT does not bind to cells, indicating that its inactivity stems from inability to engage BFTR rather than defective catalytic function. While disappointing for therapeutic development, this finding provides important mechanistic insight. BFT binding and catalytic activity may be coupled, or the inactive mutant may have adopted a conformation incompatible with receptor recognition. Alternative competitive inhibitor strategies such as receptor-blocking antibodies or small molecule antagonists warrant exploration.

In vivo validation confirmed that His-rETBF retained equivalent pathogenic potential to conventional rETBF in mouse infection models. Both strains induced identical patterns of colitis with comparable bacterial colonization levels (~1 × 10^8^ CFU/g in small intestine, ~1 × 10^9^ CFU/g in colon), demonstrating that C-terminal His-tagging does not compromise bacterial fitness, tissue tropism, or inflammatory response induction. The preserved anatomical specificity (colonic inflammation without ileal involvement) and histopathological features (epithelial shedding, crypt hyperplasia, neutrophil infiltration) validate His-rETBF as a functionally equivalent substitute for wild-type ETBF in experimental studies. However, it should be noted that both recombinant strains (His-rETBF and rETBF) induced relatively milder colitis (~80% severity) compared to wild-type ETBF strains. The underlying mechanisms responsible for this attenuated pathogenicity in recombinant strains remain unclear and warrant further investigation. Despite this limitation, the recombinant strains maintain the essential pathogenic characteristics necessary for mechanistic studies while providing the technical advantages of plasmid-based His-tag expression.

Important technical limitations warrant discussion. BFT detection and quantification relied on anti-His tag antibodies rather than anti-BFT antibodies, as BFT-specific commercial antibodies remain unavailable [25,46,47]. While this approach successfully enabled IMAC purification and Western blot detection, it prevents direct visualization of native BFT in tissues or culture supernatants without His-tag modification. Future development of high-affinity anti-BFT monoclonal antibodies would significantly enhance BFT research capabilities.

## 4. Conclusions

This study successfully developed His-recombinant type *B. fragilis* strains (His-rNTBF and His-rETBF) producing His-tagged BFT-2 (catalytically inactive form or active form), establishing the His-tagging methodology through a two-round PCR strategy combined with conjugation-based genetic transfer. This technical advancement enables high-purity hBFT purification that overcomes limitations of culture filtrate-derived native BFT and provides essential tools for BFTR identification through sophisticated biochemical approaches including pull-down assays, co-immunoprecipitation, and structural biology studies. The developed hBFT was fully characterized and demonstrated biological activity identical to native BFT despite the His-tag modification.

## 5. Materials and Methods

### 5.1. Bacterial Strains, Plasmids, and Culture Conditions

Wild-type NTBF, recombinant-type NTBF and recombinant-type ETBF (*Bacteroides fragilis* NCTC 9343) were used as parental strains for His-tagged recombinant strain construction. *Escherichia coli* DH5α was used for plasmid propagation and cloning procedures. Wild-type *E. coli* S17-1 λ pir, wild-type *E. coli* HB101 and helper plasmid pRK2013 were used for conjugative transfer of His-recombinant plasmids into *B. fragilis*. *E. coli* strains were cultured aerobically at 37 °C in Luria–Bertani (LB) broth prepared from Difco LB Broth, Miller (#244620, Becton Dickinson, Franklin Lakes, NJ, MD, USA) or on LB agar supplemented with appropriate antibiotics (ampicillin 100 μg/mL). Cells were harvested at mid-log phase (OD600 = 0.8) for all experiments. *B. fragilis* strains were cultured anaerobically at 37 °C in brain heart infusion supplemented (BHIS) broth or on BHIS agar [28,48]. Cells were harvested at mid-log phase (OD660 = 0.5–0.8) for all experiments. BHIS medium was prepared by dissolving 37 g/L Brain Heart Infusion (#MB-B1008, MB cell, Seoul, Republic of Korea), 5 g/L Bacto Yeast Extract (#212750, Becton Dickinson, Franklin Lakes, NJ, MD, USA), and 0.5 g/L L-cysteine (#168149-500G, Aldrich Chemistry, St. Louis, MO, USA) in distilled water, with addition of 15 g/L Difco Agar (#214010, Becton Dickinson, Franklin Lakes, NJ, MD, USA) for solid medium preparation. Antibiotic selection for *B. fragilis* transformants was performed using BHIS agar or broth supplemented with clindamycin 6 μg/mL and gentamicin 50 μg/mL. For conjugation experiments, selective media contained clindamycin 50 μg/mL and gentamicin 200 μg/mL on both LB agar and BHIS agar. All anaerobic cultures were maintained in an anaerobic chamber with an atmosphere of 85% N_2_, 10% H_2_, and 5% CO_2_. *Bacteroides fragilis* NCTC 9343 (strain VPI 2553) was purchased from the American Type Culture Collection (Cat# 25285, ATCC, Manassas, VA, USA). Recombinant NTBF and ETBF were obtained from previous work [49]. *Escherichia coli* strains, except for DH5α and TOP10, were kindly provided by Prof. Jeong-Soo Lee. *E. coli* DH5α was purchased from Takara Bio (#9057, Kusatsu, Shiga, Japan), and TOP10 was obtained from Cosmogenetech (Seoul, Republic of Korea).

### 5.2. Construction of C-Terminal His-Tagged bft-2 Expression Vector

The C-terminal 6× His-tagged *bft-2* expression construct was generated through two-step PCR amplification. The pFD340-*bft-2* plasmid was extracted from rETBF using the AccuPrep^®^ Plasmid Mini Extraction Kit (#K-3030-1, Bioneer Corp., Daejeon, Republic of Korea) following the manufacturer’s protocol. The extracted plasmid was used as template for first-round PCR incorporating the 6× His-tag coding sequence (CAT CAT CAC CAT CAC CAC) immediately upstream of the stop codon of *bft-2*. The first-round PCR was performed under the following conditions: Initial denaturation at 95 °C for 5 min; 30 cycles of 95 °C for 30 s, 57 °C for 30 s, and 72 °C for 30 s; followed by a final extension at 72 °C for 5 min. The amplicons (1st PP) were excised from a 1.0% agarose gel and purified using a AccuPrep^®^ PCR/Gel Purification Kit (#K-3038, Bioneer Corp., Daejeon, Republic of Korea) according to the manufacturer’s instructions. The purified first-round PCR product was used as template for second-round PCR incorporating the restriction site of *Bam*HI downstream of the stop codon. The second-round PCR was performed under the following conditions: initial denaturation at 95 °C for 5 min; 30 cycles of 95 °C for 30 s, 57 °C for 30 s, and 72 °C for 30 s; followed by a final extension at 72 °C for 5 min.

The amplicons (2nd PP) were excised from a 1.0% agarose gel and purified using a AccuPrep^®^ PCR/Gel Purification Kit. The purified second-round PCR product and plasmid were separately double-digested with restriction enzymes *Sma*I (#R015S, Enzynomics, Daejeon, Republic of Korea) and *Bam*HI (#R003S, Enzynomics, Daejeon, Republic of Korea) at 37 °C for 16 h. The double-digested 2nd PP was ligated into double-digested pFD340-*bft-2* shuttle vector using T4 DNA ligase (#E-3061, Bioneer Corp., Daejeon, Republic or Korea) under the following condition: 25 °C for 3 h and then left at 4 °C overnight (approximately 15 h). The ligated recombinant plasmid [pFD340-*bft-2*(His_6_)] was transformed into *E. coli* TOP10, DH5α, HB101, or BL21 competent cells [50]. Antibiotic selection for *E. coli* transformants was performed using LB agar supplemented with ampicillin 100 μg/mL. Positive clones were verified by colony PCR and Sanger sequencing. Transformants were stored at −80 °C in 20% glycerol stock until use. The construction of pFD340-*bft-2*(E349A-His_6_) was performed using the same procedures as described above, except that pFD340-*bft-2*(E349A) was used as the template DNA for the first-round PCR. Primer sequences are provided (Table 1).

Plasmid DNA was extracted using the AccuPrep^®^ Plasmid Mini Extraction Kit following the manufacturer’s protocol. Briefly, bacterial cells from overnight cultures were pelleted by centrifugation, resuspended in lysis buffer, neutralized, and cleared lysate was applied to spin columns. Plasmid DNA was eluted in Nuclease-free Water (#AM9937, Ambion, CA, USA) and quantified by spectrophotometry.

### 5.3. Conjugative Transfer from E. coli to B. fragilis

Conjugative transfer was performed using a biparental mating procedure. *E. coli* S17-1 λ pir harboring pFD340-*bft-2*(His_6_) (donor) and *B. fragilis* NCTC 9343 (recipient) were cultured to mid-logarithmic phase. Donor *E. coli* strain was cultured aerobically in LB broth at 37 °C for 24 h, while recipient *B. fragilis* strains were cultured anaerobically in BHIS broth at 37 °C for 48 h. Cells were harvested by centrifugation, washed twice with pre-reduced phosphate-buffered saline (PBS), and resuspended to equal optical densities. Donor and recipient cells were mixed at a ratio of 1:3 and spotted onto pre-reduced BHIS agar plates without antibiotics. Mating mixtures were incubated aerobically at 37 °C for 24 h. Following incubation, cells were resuspended in BHIS broth and plated onto selective BHIS agar containing clindamycin 50 μg/mL and gentamicin 200 μg/mL to counter-select *E. coli* strain while selecting for *B. fragilis* transconjugants. Plates were incubated anaerobically at 37 °C for 48 h until colonies appeared. Individual colonies were streaked for purification and verified by Sanger sequencing, antibiotic resistance testing, and bile esculin tolerance testing on Bacteroides bile esculin agar (BBEA).

### 5.4. Purification of hBFT-2 Variants by Immobilized Metal Affinity Chromatography

His-rNTBF and His-rETBF strains were cultured anaerobically in BHIS broth supplemented with clindamycin 6 μg/mL and gentamicin 50 μg/mL at 37 °C for 48 h. Culture supernatants were harvested by centrifugation at 14,000× *g* for 5 min at 4 °C and filtered through 0.45 μm Minisart^®^ Filter Unit (#S6555-FMOSK, Sartorius, Goettingen, Germany) to remove residual bacterial cells. For small-scale purification, filtered supernatants (up to 10 mL) were processed using the Capturem His-Tagged Purification Miniprep Kit (#635710, TaKaRa, San Jose, CA, USA) according to manufacturer’s instructions. Briefly, 800 μL of supernatants were applied to pre-equilibrated spin columns twice. Columns were washed extensively with wash buffer containing no imidazole, and His-tagged proteins were eluted with elution buffer containing 500 mM imidazole. Alternative purification was performed using the Native IMAC Buffer Kit (#6200239, Bio-Rad, Hercules, CA, USA) with gravity-flow glass columns. Filtered culture supernatants were loaded onto Ni-NTA agarose columns pre-equilibrated with native binding buffer (50 mM sodium phosphate pH 8.0, 300 mM NaCl). Columns were washed sequentially with wash buffer, and His-tagged BFT was eluted with elution buffer containing 250 mM imidazole. Imidazole removal from purified protein samples was performed by diafiltration using Amicon^®^ Ultra-0.5 mL Centrifugal Filters (Ultracel^®^-10K, #UFC501096, Merck, Carrigtwohill, Ireland). Samples were concentrated and buffer-exchanged into storage buffer (20 mM Tris-HCl pH 7.4, 150 mM NaCl, 10% glycerol) through repeated cycles of centrifugation and buffer addition according to manufacturer’s protocol. Purified proteins were quantified by Bradford assay, analyzed by SDS-PAGE and silver staining, and stored at −80 °C until use. In small-scale purification using a mini-prep format, 1301.1 µg of inactive hBFT was obtained from 25 mL of His-rNTBF-cultured BHIB, whereas 1238.63 µg of active hBFT was obtained from 25 mL of His-rETBF-cultured BHIB. For large-scale purification using a gravity flow glass column, approximately 289 µg of hBFT was obtained from 500 mL cultures of either His-rNTBF or His-rETBF.

### 5.5. Silver Staining

Protein samples separated by SDS-PAGE were visualized using the Pierce Silver Stain Kit (#24612, Thermo Scientific, Waltham, MA, USA) following the manufacturer’s protocol. Briefly, gels were fixed in fixative solution (30% ethanol, 10% acetic acid) for 30 min, washed with 10% ethanol and then Ultra Pure Water (#MS12000B, Bandio Bio Science, Pocheon, Republic of Korea) sensitized with sensitizer solution for 1 min, washed again with ultrapure water, stained with silver staining solution for 30 min in the dark, washed with ultrapure water, developed until desired band intensity appeared, and stopped with 5% acetic acid. Gels were imaged using a gel documentation system.

### 5.6. SDS-PAGE and Western Blot Analysis

Protein samples were mixed with 5× protein sample buffer (#M830000J, Bandio, Pocheon, Republic of Korea) and heated at 95 °C for 5 min. For electrophoresis, 5–10 µg of purified hBFT or 20–30 µg of total protein from cell lysates were loaded per well. Denatured samples were resolved on 10% SDS-polyacrylamide gels using a Mini-PROTEAN Tetra Cell electrophoresis system (Bio-Rad, USA) at 80 V for 20 min followed by 120 V for 120 min. Separated proteins were transferred onto nitrocellulose (NC) membranes using a wet transfer apparatus at constant 200 mA for 120 min in ice-cold transfer buffer (25 mM Tris, 192 mM glycine, 20% methanol). Membranes were blocked with 5% (*w*/*v*) skim milk in 1× Tris-buffered saline with 0.1% Tween-20 (TBST) for 1 h at room temperature with gentle shaking. Primary antibodies were diluted in 5% skim milk-TBS at a dilution of 1:2000 and incubated with membranes overnight at 4 °C with gentle shaking. The following primary antibodies were used: anti-E-cadherin (C36; #610182, Becton Dickinson, Franklin Lakes, NJ, MD, USA), anti-sE-cadherin (HECD-1; #13-1700, Invitrogen, Carlsbad, CA, USA), anti-6× His tag (His.H8; #MA1-21315, Invitrogen, USA), anti-GAPDH (6C5; #CB1001, Calbiochem, San Diego, CA, USA). After primary antibody incubation, membranes were washed three times with 1× TBST for 10 min each. Horseradish peroxidase (HRP)-conjugated secondary antibodies were diluted in 5% skim milk-TBST at a dilution of 1:10,000 and incubated with membranes for 1 h at room temperature. The following HRP-conjugated antibodies were used: Goat anti-mouse Ig (#115-035-146, Jackson ImmunoResearch, West Grove, PA, USA). Membranes were washed six times with 1× TBST and developed using SuperSignalTM West Pico PLUS Chemiluminescent Substrate (#34580, Thermo Scientific, IL, USA). Chemiluminescent signals were detected using an imaging system.

### 5.7. Cell Culture

Human colonic epithelial cell line HT29/c1 (#HTB-38, ATCC, Manassas, VA, USA; passage 130) and mouse rectal epithelial cell line CMT93 (#CCL-223, ATCC, VA, USA; passage 75) were maintained in Dulbecco’s Modified Eagle Medium (DMEM, #MD10000A, Bandio Bio Science, Pocheon, Republic of Korea) supplemented with 5% fetal bovine serum (FBS, #35-015-CV, Corning, Palo Alto, CA, USA), 20 mM HEPES (#15630-080, Gibco, Miami, FL, USA), without penicillin-streptomycin [51,52]. Mouse small intestinal epithelial cell lines MSIE (passage 20) and YAMC (passage 52) were maintained in DMEM supplemented with 10% FBS and 20 mM HEPES. All cells were cultured at 37 °C in a humidified atmosphere containing 5% CO_2_. Cell lines were routinely tested for mycoplasma contamination and authenticated by morphology and growth characteristics. For subculturing, all cells were resuspended in 0.25% trypsin-EDTA (TE, #25200-072, Gibco, Birlington, ON, Canada) buffer and neutralized with serum DMEM. The MSIE and YAMC cell lines were kindly provided by Dr. Eugene Chang (University of Chicago).

### 5.8. RNA Extraction and Reverse Transcription-Quantitative PCR (RT-qPCR)

Total RNA was extracted from bacterial cultures or mammalian cells using TRIzolTM Reagent (#15596026, Invitrogen, CA, USA) according to manufacturer’s instructions. Briefly, samples were homogenized in TRIzol, mixed with 1/5-fold volume of chloroform, and centrifuged to separate phases. The aqueous phase containing RNA was precipitated with identical volume of isopropanol, washed with 75% ethanol, air-dried, and dissolved in DEPC-treated water (#MS17000C, Bandio, Pocheon, Republic of Korea). RNA concentration and purity were assessed by spectrophotometry (A260/A280 ratio > 1.8). Complementary DNA (cDNA) was synthesized from 1–2 μg of total RNA using following reagents: Random Primers (#58875, Invitrogen, CA, USA), Oligo (dT) 12–18 Primer (#18418012, Invitrogen, MD, USA), dNTP (#BDN-9140, Tech & Innovation, Chuncheon, Republic of Korea), 5× First Strand Buffer (#y02321, Invitrogen, USA), 0.1M DTT (#y00147, Invitrogen, Carlsbad, CA, USA), M-MLV Reverse Transcriptase (#28025-021, Invitrogen, USA).

Conventional PCR was performed using Prime Taq DNA Polymerase and Prime Taq Premix (#G-1000, GenetBio, Daejeon, Republic of Korea). Quantitative real-time PCR was performed using 2× SYBR^®^ Green PCR Master Mix (#4309155, Applied Biosystems, Carlsbad, CA, USA) on a real-time PCR system (QuantStudioTM 1 Real-Time PCR Instrument 96-Well 0.2 mL Block, #A40425, Thermo Scientific, Waltham, MA, USA). Primer sequences for target genes were custom-synthesized by Cosmogenetech (Seoul, Republic of Korea) [53]. Cycling conditions consisted of initial denaturation at 95 °C for 10 min, followed by 40 cycles of 95 °C for 15 s and 55–60 °C for 1 min. Melting curve analysis and agarose gel electrophoresis were performed to verify amplicon specificity. Relative gene expression was calculated using the 2^−ΔΔCt^ method with normalization to housekeeping genes (16S rRNA for bacterial samples and GAPDH for mammalian cells).

### 5.9. Confocal Microscopy

Cells were seeded onto confocal dishes (#101350, SPL Life Sciences, Pocheon, Republic of Korea) and cultured until 60–70% confluence. Following experimental treatments, cells were fixed with 4% paraformaldehyde (PFA) in PBS for 15 min at room temperature, washed three times with PBS, and permeabilized with ice-cold 100% methanol for 10 min at −20 °C. After washing with PBS, cells were blocked with 1% bovine serum albumin (BSA) in Dulbecco’s phosphate-buffered saline (DPBS) for 1 h at room temperature. Primary antibodies were diluted in 1% BSA-DPBS and incubated with cells overnight at 4 °C in a humidified chamber. After washing three times with DPBS, Alexa Fluor 488-conjugated goat anti-mouse IgG secondary antibody (#A11001, Invitrogen, OR, USA) was diluted in 1% BSA-DPBS and incubated for 1 h at room temperature in the dark. Cells were washed three times with DPBS, and nuclei were counterstained with VECTASHIELD^®^ Antifade Mounting Medium with DAPI (#H-1200, Vector Laboratories, Newark, CA, USA). Confocal images were acquired using a Zeiss LSM 710 confocal laser scanning microscope (Carl Zeiss, Germany) at the MIRAE Campus of Yonsei University or a Zeiss LSM 800 confocal microscope at Wonju Severance Christian Hospital. Z-stack images were obtained with optimal section thickness, and images were processed using ZEN 2.5 software (Carl Zeiss, Oberkochen, Germany) and ImageJ (version 1.54r).

### 5.10. Animal Experiments

Female C57BL/6 mice (10 weeks old, 18–22 g) were purchased from Orient Bio (Seongnam, Republic of Korea) and housed in specific pathogen-free conditions with ad libitum access to food and water under 12 h light/dark cycles. All animal experiments were conducted in accordance with institutional guidelines. For ETBF infection experiments, mice were pretreated with antibiotics by providing drinking water containing clindamycin 100 μg/mL and gentamicin 300 μg/mL for 3–5 days to deplete normal intestinal microbiota and facilitate ETBF colonization. Antibiotic-containing water was replaced every 2 days. Following antibiotic pretreatment, mice were orally inoculated with WT-ETBF, His-rETBF or rETBF (≥1 × 10^10^ CFU in 100 μL of sterile PBS) by gavage using a feeding needle. Control groups received sterile PBS or were inoculated with His-rNTBF. Mice were monitored daily for body weight changes, stool consistency, and clinical signs of disease. At designated time points, mice were euthanized by CO_2_ asphyxiation, and intestinal tissues (ileum, cecum, colon) were harvested for subsequent analyses.

### 5.11. Histology and Hematoxylin-Eosin (H&E) Staining

Intestinal tissue samples were fixed in 10% neutral-buffered formalin for 24 h, dehydrated through graded ethanol series, cleared in xylene, and embedded in paraffin wax to generate formalin-fixed paraffin-embedded (FFPE) blocks. Tissue sections (4 μm thickness) were cut using a microtome (model, company, country), mounted onto glass slides, and dried overnight at 37 °C. For H&E staining, sections were deparaffinized in xylene, rehydrated through graded ethanol to distilled water, stained with hematoxylin for 5 min, differentiated in 1% HCl-alcohol, blued in DPBS, counterstained with eosin Y for 4 min, dehydrated through graded ethanol, cleared in xylene, and mounted with xylene mounting medium Optic MountTM X (##7722MIRA01-4OZ, BBC Biochemical, Mount Vernon, WA, USA). Stained sections were examined under a light microscope (#DM 2500, Leica, Singapore) and images were captured using imaging software. Histological scoring of inflammation was performed by a blinded pathologist according to established criteria assessing epithelial damage, inflammatory cell infiltration, and mucosal architecture disruption.

### 5.12. Statistical Analysis

All experiments were performed with at least three independent biological replicates unless otherwise stated. Data are presented as mean ± standard error of the mean (SEM) or median ± 95% confidence interval as indicated in figure legends. Data distribution was assessed for normality using the Shapiro–Wilk test prior to performing parametric statistical analyses. Statistical comparisons between two groups were performed using unpaired Student’s *t*-test. Comparisons among multiple groups were performed using one-way analysis of variance (ANOVA) followed by Tukey’s post hoc test for pairwise comparisons. Statistical significance was defined as *p* < 0.05. All statistical analyses were performed using GraphPad Prism software (v.9.0, GraphPad Software, Boston, MA, USA).

## Figures and Tables

**Table 1:** Primer sequences used in this study.

Experimental Usage	Target Gene		Sequence (5′-3′)	Ta (°C)	Amplicon Size (bp)
Cloning/1st PCR	pFD340-*bft-2* pFD340-*bft-2*(E349A)	F	CCA GCT GAT GTA TCC CGG G	57	177
		R	GTG GTG ATG GTG ATG ATG ATC GCC ATC TGC TAT TTC CC		
Cloning/2nd PCR	1st PCR product	F	CCA GCT GAT GTA TCC CGG G	57	232
		R	TGA CTC TAG AGG ATC CAT TAA TCG AAC TTC GAT TCT CAC TCT TTG GTT ATC TAG TGG TGA TGG TGA TGA TGA TC		
RT-qPCR	Mouse *cxcl1*/*kc*	F	TCC AGA GCT TGA AGG TGT TGC C	55	200
		R	AAC CAA GGG AGC TTC AGG GTC A		
	Mouse *gapdh*	F	TGA GCA AGA GAG GCC CTA TC	55	94
		R	AGG CCC CTC CTG TTA TTA TG		
	Human *cxcl8*/*il-8*	F	GAG AGT GAT TGA GAG TGG ACC AC	55	200
		R	CAC AAC CTT CTG CAC CCA GTT T		
	Human *gapdh*	F	CGG GAA GCT TGT CAT CAA TGG	55	349
		R	GGC AGT GAT GGC ATG GAC TG		

## Data Availability

The original contributions presented in this study are included in the article. Further inquiries can be directed to the corresponding author.

## Abbreviations

The following abbreviations are used in this manuscript:

*B. fragilis*	*Bacteroides fragilis*
ETBF	Enterotoxigenic *B. fragilis*
BFT	*B. fragilis* toxin
BFTR	Cellular receptor for BFT (BFT receptor)
hBFT	His-tagged recombinant BFT
A-hBFT	Catalytically active hBFT
I-hBFT	Catalytically inactive mutant hBFT
SFM	Serum-free medium
DAPI	4′,6-diamidino-2-phenylindole
MSIE	Mouse small intestine epithelial (cell line)
YAMC	Young adult mouse colon (cell line)
